# Improving Spatiotemporal Breast Cancer Assessment and Prediction in Hangzhou City, China

**DOI:** 10.1038/s41598-017-03524-z

**Published:** 2017-06-09

**Authors:** Zhaohan Lou, Xufeng Fei, George Christakos, Jianbo Yan, Jiaping Wu

**Affiliations:** 1 Institute of Islands and Coastal Ecosystems, Zhejiang University, Zhoushan, China; 2 College of Environmental and Resource Sciences, Zhejiang University, Hangzhou, China; 3 Department of Geography, San Diego State University, San Diego, CA USA; 4 Zhoushan Center for Disease Control and Prevention, Zhoushan, China

## Abstract

Breast cancer (BC) is the main cause of death of female cancer patients in China. Mainstream mapping techniques, like spatiotemporal ordinary kriging (STOK), generate disease incidence maps that improve our understanding of disease distribution. Yet, the implementation of these techniques experiences substantive and technical complications (due mainly to the different characteristics of space and time). A new spatiotemporal projection (STP) technique that is free of the above complications was implemented to model the space-time distribution of BC incidence in Hangzhou city and to estimate incidence values at locations-times for which no BC data exist. For comparison, both the STP and the STOK techniques were used to generate BC incidence maps in Hangzhou. STP performed considerably better than STOK in terms of generating more accurate incidence maps showing a closer similarity to the observed incidence distribution, and providing an improved assessment of the space-time BC correlation structure. In sum, the inter-connections between space, time, BC incidence and spread velocity established by STP allow a more realistic representation of the actual incidence distribution, and generate incidence maps that are more accurate and more informative, at a lower computational cost and involving fewer approximations than the incidence maps produced by mainstream space-time techniques.

## Introduction

Accounting for almost 25% of all female cancer cases and 15% of all female cancer deaths in the world, breast cancer (BC) is the most common malignant tumor among females and the main cause of death of female cancer patients^1^. BC is one of the most frequently diagnosed female malignant tumors in China, with an age-standardized incidence rate of about 30/100,000^2^, and it is expected to account for 15% of all new cancers in women from 2009 to 2011^3^. Moreover, BC incidence in China keeps growing in recent years, especially in sub-town and rural areas. During the period 2000–2011, BC incidence in China kept an increasing trend with an annual percentage change (APC) of 3.9^3^. BC, being one of the most important public health issues worldwide, many of its risk factors have been studied by scientists, such as genetic susceptibility^4^, diet and alcohol consumption^5, 6^, body mass index (BMI)^7^, reproductive, menstrual and hormonal factors^8–11^.

Geostatistical kriging techniques are highly successful spatial data analysis and estimation techniques used in numerous scientific disciplines^12^. Several previous studies have analyzed the distribution of BC at the local and at the global scale^5, 13^, and they found that the BC variability differed between developed and developing regions. Few studies have used kriging techniques to estimate BC incidence based on a small sample of case data. To meet the demands of space-time data analysis and estimation, spatiotemporal kriging techniques have been proposed and the associated spatiotemporal covariance (or variogram) modeling has been developed^14^. The selection of adequate theoretical covariance models to represent the space-time distribution of disease incident and the accurate specification of the model parameters are the keys of a realistic space-time disease representation. A useful classification of space-time covariance models distinguishes between separable and non-separable models^15^. The group of separable models includes the additive (linear) and the multiplicative (product) models, which assume that spatiotemporal disease correlation is represented by the sum or the product, respectively, of a spatial and a temporal component^16^, thus making model parameter estimation easy and fast. The non-separable group of covariance models, which includes the product-sum, the metric and the sum-metric models^17^, in certain cases can describe better the space-time structure of disease data. However, it is usually hard to specify the parameters of non-separable covariance models based on the available data. By comparison, spatial estimation techniques (like ordinary and indicator krigings^12^), being around much longer than their recently developed space-time counterparts, are much more developed computationally and workable in a large number of popular software libraries. Therefore, an approach that transforms spatiotemporal (*R* ^*2*^ × *T*) data analysis and estimation into spatial (*R* ^*2*^) data analysis and estimation would be a particularly welcomed development, potentially improving modeling and estimation accuracy as well as computational efficiency. Such an approach, the so-called space-time projection (STP) technique, has been recently developed^18^.

Accordingly, the objective of this work is twofold: (1) use the novel STP technique to study BC incidence in Hangzhou city during the period 2008–2012 (which is the first time that STP is used for this kind of non-infectious disease); and (2) compare the space-time BC incidence estimates and maps generated by the STP technique with those obtained by the mainstream STOK technique (in terms of map accuracy, model efficiency, and computational effort).

## Materials and Methods

### Study area

**Figure 1:**
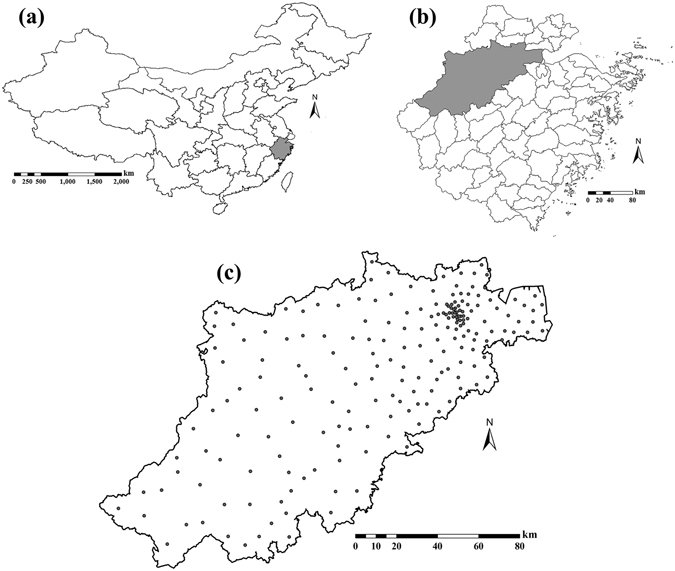
BC incidence data locations in Hangzhou city during the period 2008–2012. (**a**) Chinese provinces boundaries; (**b**) city boundaries in Zhejiang province; (**c**) Hangzhou city and BC incidence locations. Maps were carried out with ArcGIS 9.3. URL link: http://desktop.arcgis.com/zh-cn/desktop/.

Hangzhou city is located in the southeast coastal region of China (E 118°21′–120°30′, N 29°11′–30°33′), and is the capital city of Zhejiang province including 200 townships (towns and subdistricts), Fig. 1. Hangzhou covers a territory of about 16,596 *km* ^2^, and its total population is about 8.70 million according to the 6^th^ national population census (2010). Hangzhou has a typical subtropical monsoon climate, with four distinct seasons, plenty of sunshine and abundant rainfall. The northeastern part of Hangzhou belongs to the plain areas of northern Zhejiang with low elevation, fertile soil, variegated rivers and plentiful natural resources, thus making it suitable for habitation and city development. The southwestern part of Hangzhou belongs to the hilly area of western Zhejiang Province characterized by high elevations and a large area with forests and mountains, which means that this part is less polluted and economically underdeveloped. According to the Chinese Cancer Registry Annual Report (2012), Hangzhou was one of the cities with the highest BC incidence, having 1700 newly diagnosed female BC cases. The age-standardized incidence rate (adjusted for world population) was about 33.63/100,000 in 2009^19^.

### Data set

**Figure 2:**
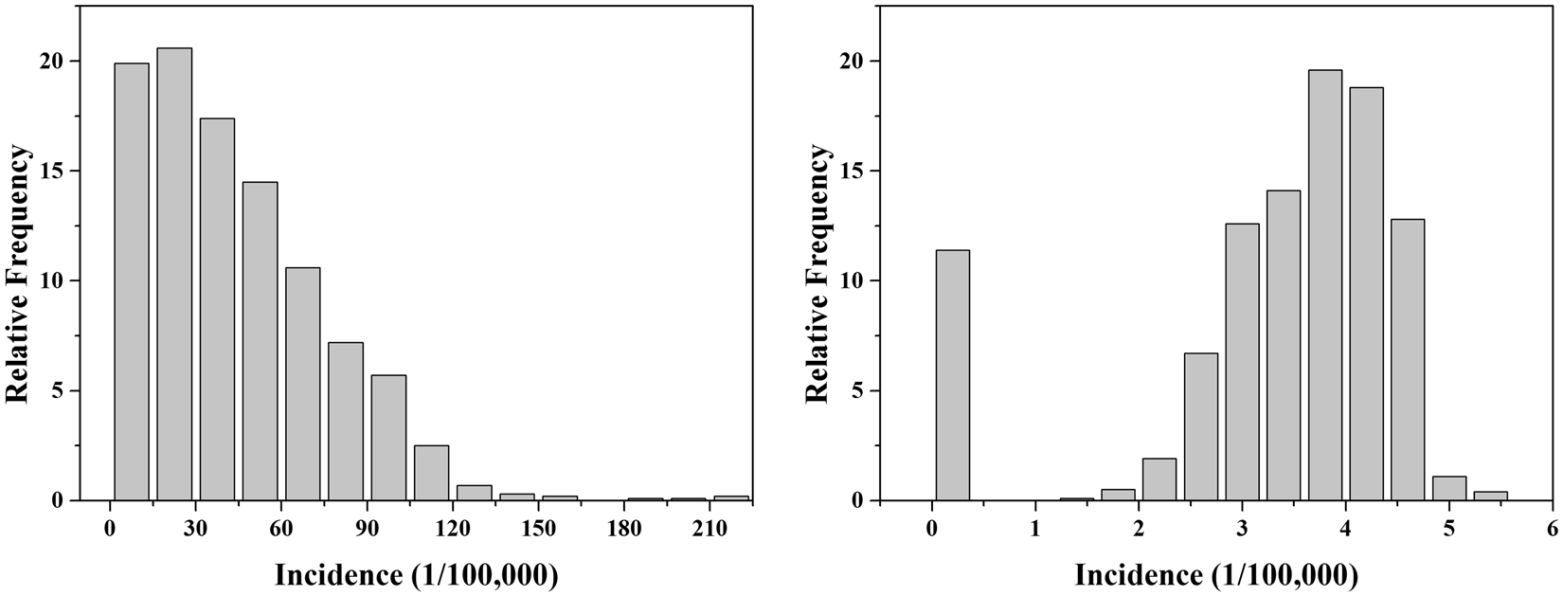
(**a**) BC incidence data histogram and (**b**) BC incidence data histogram after the data have been transformed as $\mathrm{log}(BC+1)$.

Anonymized female BC patient records (International Classification of Disease: ICD-10: C50) were obtained from the Center of Disease Control and Prevention (CDC). Cancer data in Hangzhou was registered through the International Association of Cancer Registries (IACR) recommended software CanReg4 and checked by the Chinese National Cancer Center to ensure data reliability. In briefly, 8784 BC cases in total (1643 in 2008, 1727 in 2009, 1820 in 2010, 1812 in 2011 and 1782 in 2012) were diagnosed in Hangzhou during the period 2008–2012. According to the detailed household register information, all these cases were allocated into 200 townships. An indirect standardization method^20^, which can control the difference caused by heterogeneous age structure, was used to calculate the age-standardized incidence based on (*a*) the female age-specific population data at the township level obtained from the 6^th^ national population census and (*b*) the most resent Chinese female age specific BC incidence obtained from the Chinese National Cancer Center^19^. The geometric centers of the 200 townships were used to denote their locations. The locations and histogram of BC incidence records in the 200 townships are shown in Fig. 1c and Fig. 2, respectively. Summary BC incidence statistics is presented in Table 1. There are 1000 BC incidence cases in total at township level, ranging from 0.00 to 111.72/100,000 with a mean of 42.06/100,000, a standard deviation of 28.55/100,000 and a CV of 67.88%. Considering the left-skewness of original data histogram (Fig. 2a), BC incidence was natural log(*BC* + 1)-transformed to follow a normal distribution (Fig. 2b). Previous studies have shown that the BC incidence distribution was spatially heterogeneous in Hangzhou (revealing an increasing incidence trend from the southern to the northern parts of the city) and temporally stable during the period 2008–2012^11, 21^, thus making it a suitable data set to be studied by the STOK and the STP techniques for the purpose of space-time modeling, estimation and mapping of the BC incidence distribution.

**Table 1:** Summary statistics of BC incidence (per 100,000 cases).

	Number	Min	Max	Mean	SD^a^	C^b^
*BC data set*	1000	0.00	111.72	42.06	28.55	67.88%

^a^Standard deviation.

^b^Coefficient of variation.

### The Spatiotemporal BC Traveling Model

Considering that BC incidences vary across both space and time (composite space-time BC incidence distribution) under conditions of uncertainty, the spatiotemporal random field (S/TRF) theory^14^ was used to describe the statistical properties of the space-time BC incidence distribution. This distribution is represented mathematically by the random field *BC*(***s***, *t*), where (***s***, *t*) = (*s*_1_, *s*_2_, *t*) ∈ *R* ^2^ × *T* denote points in space and time, including the geographical coordinates ***s*** = *s*_1_, *s*_2_ and time instant *t*. Methodologically, the random field *BC*(***s***, *t*) is viewed as a collection of realizations (possibilities) of the BC incidence distribution, where the probability that each one of these realizations occurs is expressed by the BC incidence probability law (Gaussian or non-Gaussian). This BC model makes it possible to calculate various space-time properties of incidence distribution with reasonable accuracy^11, 22^. In particular, the *BC*(***s***, *t*) incidence distribution is represented by^18^

1 $$BC({\boldsymbol{s}},t)=BC({\boldsymbol{s}}-{\boldsymbol{\upsilon }}t,0)=BC(\mathop{{\boldsymbol{s}}}\limits^{{\boldsymbol{\frown }}{}}),$$

where $BC(\mathop{{\boldsymbol{s}}}\limits^{{\boldsymbol{\frown }}{}})$ is the so-called travelling random field model of BC incidence, and $\mathop{{\boldsymbol{s}}}\limits^{{\boldsymbol{\frown }}{}}={\boldsymbol{s}}-{\boldsymbol{\upsilon }}t\in {R}^{2}$. The vector ***υ ***= (*υ*_1_, *υ*_2_) describes the velocity (direction and magnitude or speed) of the BC incidence spread, linking the BC incidence distribution *BC*(***s***, *t*) in the three-dimensional (*R* ^2^ × *T*) domain with the travelling BC incidence distribution $BC(\mathop{{\boldsymbol{s}}}\limits^{{\boldsymbol{\frown }}{}})$ in the two-dimensional (*R* ^2^) domain. The corresponding BC incidence covariances satisfy the relationship

2 $${c}_{BC}({\boldsymbol{h}},\tau )={c}_{BC}(r-\upsilon \tau ,0)={c}_{BC}(\mathop{r}\limits^{\frown {}}),$$

where *c*_*BC*_(***h***, *τ*) is the spatiotemporal BC incidence covariance at the spatial lag ***h*** and time separation ***τ***, (***h***, *τ*) = (***s*** − ***s***′, *t* − *t*′) ∈ *R* ^2^ × *T*, ${c}_{BC}(\mathop{r}\limits^{\frown {}})$ is the covariance of the travelling BC incidence distribution, $\upsilon =|{\boldsymbol{\upsilon }}|=\sqrt{{\upsilon }_{1}^{2}+{\upsilon }_{2}^{2}}$, $r=|{\boldsymbol{h}}|=\sqrt{{h}_{1}^{2}+{h}_{2}^{2}}$ and $\mathop{r}\limits^{\frown {}}=r-\upsilon \tau \in {R}^{1}$. Equations (1)–(2) establish the necessary quantitative relationships between the original BC incidence data in the original *R* ^2^ × *T* domain and the traveling BC incidence data in the *R* ^2^ domain. Since Eq. (2) is derived directly from Eq. (1)^23^, the same vector ***υ*** that satisfies Eq. (1) of the incidence distribution will also satisfy Eq. (2) of the incidence covariance change, and vice versa. In practice, given the available data set, a vector ***υ*** with components *υ*_1_ and *υ*_2_ is sought so that Eqs (1)–(2) are satisfied. Then, the magnitude (speed) |***υ***| of the traveling velocity vector ***υ*** may be interpreted as representing the strength of the composite space-time correlation (dependence) of the BC incidence values along the direction of ***υ***.

Since using Eqs (1)–(2) the *BC*(***s***, *t*) can be transformed into $BC(\mathop{{\boldsymbol{s}}}\limits^{{\boldsymbol{\frown }}{}})$, we can model and estimate BC incidence in the two-dimensional domain (*R* ^2^) instead of the three-dimensional domain (*R* ^2^ × *T*), thus avoiding the complexities associated with the *R* ^2^ × *T* domain and obtaining more accurate BC incidence estimates at a lower computational cost. Subsequently, we can backtransform the BC incidence values obtained in the *R* ^2^ domain, $BC(\mathop{{\boldsymbol{s}}}\limits^{{\boldsymbol{\frown }}{}})$, into the corresponding BC incidence values in the original *R* ^2^ × *T* domain, *BC*(***s***, *t*). More specifically, since the incidence velocity vector ***υ*** and the space-time point vector (***s***, *t*) are inter-dependent and specified in a self-consistent manner, to each (***s***, *t*) of the BC incidence distribution *BC*(***s***, *t*) we can associate a unique ***υ***. This means that if we let the spatiotemporal BC field “travel” along the ***υ***-direction at a distance |***υ***|*t*, we can determine the travelling random field $BC(\mathop{{\boldsymbol{s}}}\limits^{{\boldsymbol{\frown }}{}})$ representing the BC incidence distribution. For illustration, if a high incidence region is detected in the study moving from an urban to a rural area, it implies that the high incidence region travels along the ***υ***-direction at a distance |***υ***|*t* without significant change. In this way, each point of the spatiotemporal BC incidence distribution with coordinates (*s*_1_, *s*_2_, *t*) can be “projected” into a point of the traveling BC distribution with coordinates $({\mathop{s}\limits^{\frown {}}}_{1},{\mathop{s}\limits^{\frown {}}}_{2})=({s}_{1}-\upsilon t,{s}_{2}-\upsilon t)$. This is why this approach is also called the space-time projection (STP) technique.

For data normalization purposes, the $\mathrm{log}(BC+1)$-transformed BC incidence data values were detrended with a 100,000 m spatial radius and a 2-year time radius. The spatiotemporal empirical BC incidence covariance, denoted as ${\hat{c}}_{BC}({\boldsymbol{h}},\tau )$ was made using a maximum spatial correlation range *ε*_*s*_ = 50 *km* and a maximum temporal correlation range *ε*_*t *_= 5 *yrs*. The theoretical space-time multiplicative separable covariance model of BC incidence distribution

3 $${c}_{BC}({\boldsymbol{h}},\tau )={c}_{0}{e}^{-\frac{3|{\boldsymbol{h}}{|}^{2}}{{\alpha }_{s}^{2}}-\frac{3\tau }{{\alpha }_{t}}}$$

(*c*_0_ = 1, *α*_*s*_ = 10 *km* and *α*_*t*_ = 2 *yrs*) was fitted to the computed empirical covariance ${\hat{c}}_{BC}({\boldsymbol{h}},\tau )$ (this model’s separability actually makes computer software interpolation particularly easy). In technical terms, the Gaussian spatial component of the *c*_*BC*_(***h***, *τ*) model of Eq. (3) combined with the exponential temporal component were used to jointly minimize the well-known Akaike Information Criterion (AIC)^24^, thus achieving an optimal fit.

An noted earlier, the interdependence of ***υ***, ***h*** and *τ* makes it possible to calculate the velocity vector ***υ*** from Eq. (2). More specifically, using the theoretical covariance model of Eq. (3), Eq. (2) gives

4 $${c}_{0}{e}^{-\frac{3|{\boldsymbol{h}}{|}^{2}}{{\alpha }_{s}^{2}}-\frac{3\tau }{{\alpha }_{t}}}={c}_{0}{e}^{-\frac{3{(r-\upsilon \tau )}^{2}}{{\alpha }_{s}^{2}}}={c}_{0}{e}^{-\frac{3{(|{\boldsymbol{h}}|-\upsilon \tau )}^{2}}{{\alpha }_{s}^{2}}}$$

which, after equating the exponents, gives the *υ*-equation

5 $${\alpha }_{t}\tau {\upsilon }^{2}-2{\alpha }_{t}|{\boldsymbol{h}}|\upsilon -{\alpha }_{s}^{2}=0.$$

**Figure 3:**
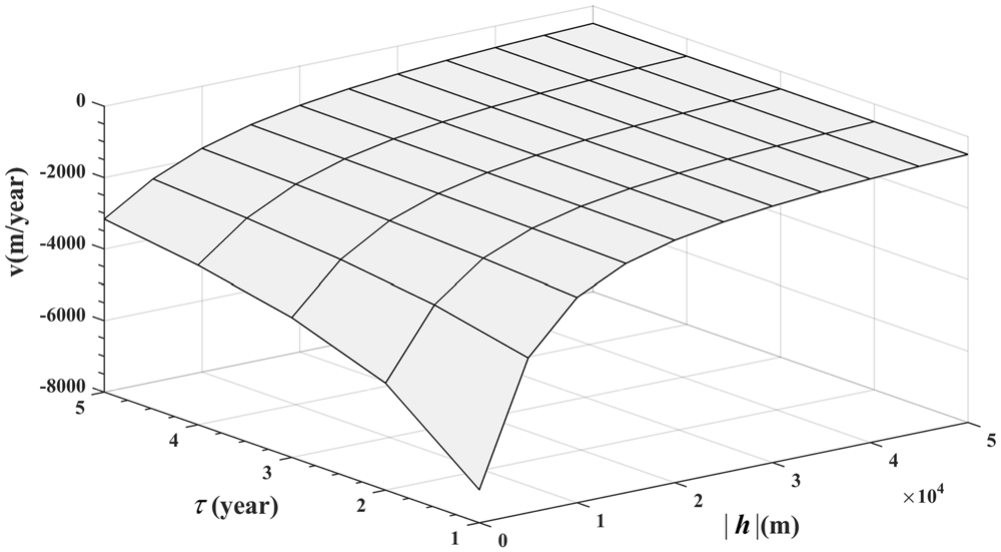
Plot of the BC incidence spread velocity (in m/year) as a function of space lag, |***h***| and time lag *τ*.

The particular solution of Eq. (5) with respect to *υ* that maintains a (physically meaningful) positive $\mathop{r}\limits^{\frown {}}$, is

6 $$\upsilon =\frac{|{\boldsymbol{h}}|}{\tau }-\frac{{\alpha }_{s}}{\tau }{[\frac{|{\boldsymbol{h}}{|}^{2}}{{\alpha }_{s}^{2}}+\frac{\tau }{{\alpha }_{t}}]}^{\frac{1}{2}},$$

which was chosen as the magnitude (speed) of the vector ***υ***, whereas the direction of vector ***υ*** was determined by vector ***h***. Using the *υ* of Eq. (6), we indeed find that

7 $$\mathop{r}\limits^{\frown {}}=r-\upsilon \tau ={\alpha }_{s}{[\frac{|{\boldsymbol{h}}{|}^{2}}{{\alpha }_{s}^{2}}+\frac{\tau }{{\alpha }_{t}}]}^{\frac{1}{2}} > 0,$$

as physically required. From a disease distribution perspective, the BC incidence field “travels” in space with spread speed |***υ***| along the direction of the spatial lag vector ***h***, that is, the |***υ***| measures the strength of the composite space-time correlation of BC incidence values along the specified direction. The distribution of the velocity vector ***υ*** is plotted in Fig. 3. With the help of ***υ***, each pair of (***s***, *t*) in *R* ^2^ × *T* is related to a unique pair of $(\mathop{{\boldsymbol{s}}}\limits^{{\boldsymbol{\frown }}{}},\,{\boldsymbol{\upsilon }})$ in *R* ^2^ through the equation $\mathop{{\boldsymbol{s}}}\limits^{{\boldsymbol{\frown }}{}}={\boldsymbol{s}}-{\boldsymbol{\upsilon }}t$ leading to the traveling incidence distribution $BC(\mathop{{\boldsymbol{s}}}\limits^{{\boldsymbol{\frown }}{}})$. For normalization purposes, the $\mathrm{log}(BC+1)$-transformed $BC(\mathop{{\boldsymbol{s}}}\limits^{{\boldsymbol{\frown }}{}})$ incidence data values were detrended with a 100 *km* spatial radius. The computation of the empirical covariance of $BC(\mathop{{\boldsymbol{s}}}\limits^{{\boldsymbol{\frown }}{}})$ was made using a maximum spatial correlation range *ε*_*s*_ = 50 *km*. An exponential theoretical covariance model was fitted to the above empirically calculated covariance as follows,

8 $${c}_{BC}(\mathop{r}\limits^{\frown {}})={c}_{BC}(r-\upsilon \tau ,0)=c{e}^{-\frac{3\mathop{r}\limits^{\frown {}}}{\alpha }},$$

where *c* = 1, *α* = 15 *km*. For BC incidence estimation purposes, the $BC(\mathop{{\boldsymbol{s}}}\limits^{{\boldsymbol{\frown }}{}})$ data set was also randomly divided into 10 groups, so that each time 9 data groups were used to estimate the remaining data group. Finally, in light of Eq. (1), the original $\mathop{BC}\limits^{\wedge }$ (***s***, *t*) estimate at an unobserved space-time point (***s***, *t*) in *R* ^2^ × *T* is related to traveling BC incidence estimate $\mathop{BC}\limits^{\wedge }(\mathop{{\boldsymbol{s}}}\limits^{{\boldsymbol{\frown }}{}})$ at the corresponding location $\mathop{{\boldsymbol{s}}}\limits^{{\boldsymbol{\frown }}{}}$ in *R* ^2^ by

9 $$\mathop{BC}\limits^{\wedge }({\boldsymbol{s}},t)=\mathop{BC}\limits^{\wedge }(\mathop{{\boldsymbol{s}}}\limits^{{\boldsymbol{\frown }}{}}),$$

where $\mathop{{\boldsymbol{s}}}\limits^{{\boldsymbol{\frown }}{}}={\boldsymbol{s}}-{\boldsymbol{\upsilon }}t$. Equation (9) allows us to generate estimates $\mathop{BC}\limits^{\wedge }$(***s***, *t*) of the BC incidence distribution in the original *R* ^2^ × *T* domain from the estimates $BC(\mathop{{\boldsymbol{s}}}\limits^{{\boldsymbol{\frown }}{}})$ in the transformed *R* ^2^ domain. We notice that, because of its reduced dimensionality, it is much easier and accurate: (*a*) to calculate an empirical BC covariance in *R* ^2^ that is a valid representative of the actual BC incidence variation, (*b*) to select a model ${c}_{BC}(\mathop{r}\limits^{\frown {}})$ and determine its parameters so that it has the best fit to the empirical BC covariance, and, finally, (*c*) to implement a computationally much faster incidence estimation technique.

In view of the above considerations, the space-time projection (STP) technique of BC incidence estimation based on Eqs (1)–(9) consists of the following steps:

i. computation of the empirical *BC*(***s***, *t*) covariance in *R* ^2^ × *T* based on the original BC incidence data, and selection of the *c*_*BC*_(***h***,*τ*) model fitted to the empirical incidence covariance;
ii. calculation of the BC velocity vector ***υ*** using Eq. (6) that connects the BC covariances in the *R* ^2^ × *T* and *R* ^2^ domains;
iii. derivation of the traveling $BC(\mathop{{\boldsymbol{s}}}\limits^{{\boldsymbol{\frown }}{}})$ incidence values in *R* ^2^ via the space-time coordinate transformation $\mathop{{\boldsymbol{s}}}\limits^{{\boldsymbol{\frown }}{}}={\boldsymbol{s}}-{\boldsymbol{\upsilon }}t$;
iv. plot of the empirical $BC(\mathop{{\boldsymbol{s}}}\limits^{{\boldsymbol{\frown }}{}})$ covariance in *R* ^2^, and selection of the corresponding ${c}_{BC}(\mathop{r}\limits^{\frown {}})$ model;
v. estimation of the BC distribution in the traveling *R* ^2^ domain, generating the $\mathop{BC}\limits^{\wedge }(\mathop{{\boldsymbol{s}}}\limits^{{\boldsymbol{\frown }}{}})$ estimation map; and, lastly,
vi. estimation of the BC distribution in the original *R* ^2^ × *T* domain using Eq. (9), thus, plotting the final $\mathop{BC}\limits^{\wedge }$(***s***, *t*) estimation map.

It should be noticed that the *R* ^2^-domain of STP data analysis is rather “pseudo-spatial” and not “purely spatial” in the conventional sense, since the spatial coordinates of STP, $\mathop{{\boldsymbol{s}}}\limits^{{\boldsymbol{\frown }}{}}={\boldsymbol{s}}-{\boldsymbol{\upsilon }}t$, include temporal incidence information (via the term ***υ*** *t*), whereas the spatial coordinates, ***s***, of the conventional purely spatial analysis (e.g., spatial statistical regression or kriging) do not include temporal incidence information.

For comparison purposes, the STOK technique was also employed in the present work to produce space-time BC incidence maps of Hangzhou city using the same original BC data set and covariance model of Eq. (3) as the STP technique. Just as was done with the STP technique, the original BC incidence data set was randomly divided into 10 groups, so that each time 9 data groups were used to estimate the remaining data group. The BC incidence estimation using STOK used a maximum number of *N* = 50 data at surrounding points, a spatial correlation range *ε*_*s*_ = 10 *km*, and a temporal range *ε*_*t*_ = 2 *yrs*.

The SEKS-GUIv1.0.8 software library^25^ was used to estimate BC incidence values of both the spatiotemporal incidence distribution *BC*(***s***, *t*) and the traveling incidence distribution $BC(\mathop{{\boldsymbol{s}}}\limits^{{\boldsymbol{\frown }}{}})$. All data processing and mapping operations were carried out with ArcMap 9.3^26^ (URL link: http://desktop.arcgis.com/zh-cn/desktop/). Other figures, like histograms, were generated using Matlab R2014b^27^ software.

## Results

**Figure 4:**
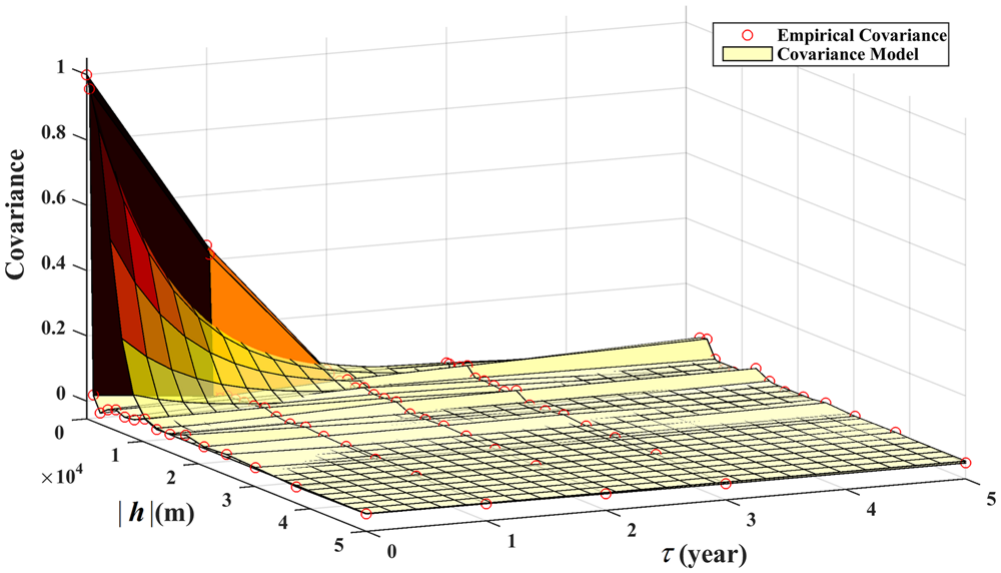
Empirical covariance (red circles) and theoretical covariance model (continuous line) of the original space-time BC incidence distribution in *R* ^2^ × *T*.

**Figure 5:**
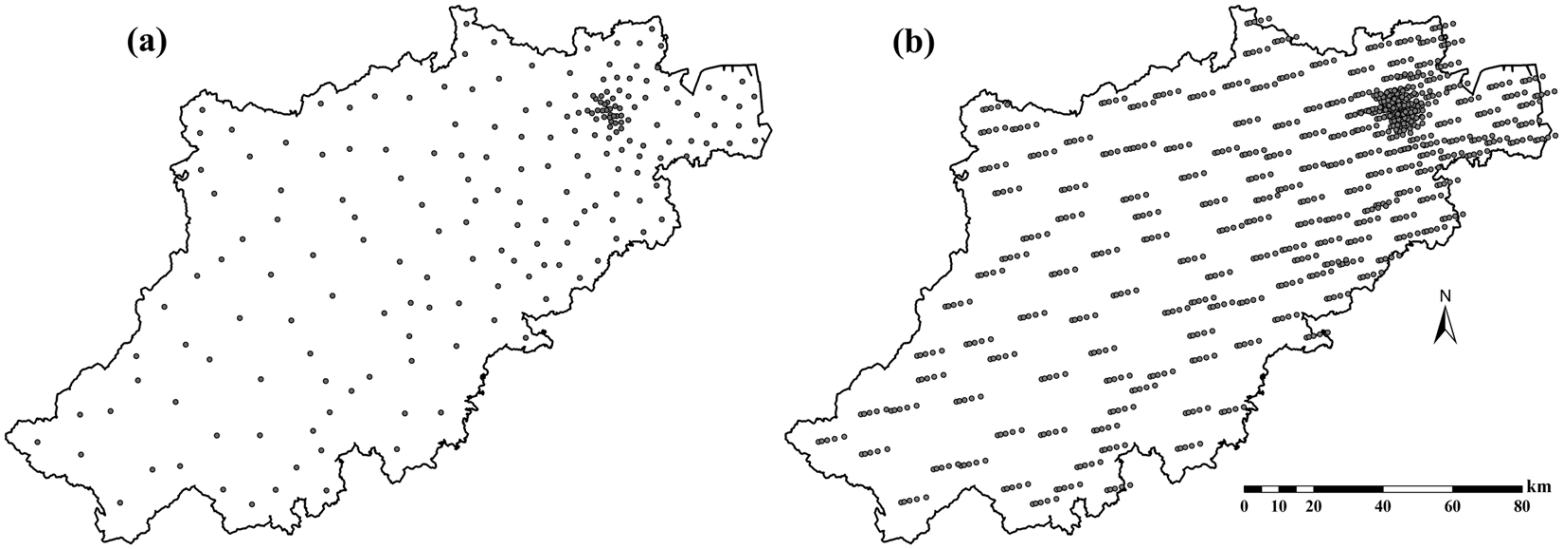
(**a**) Distribution of original BC incidence locations ***s*** in Hangzhou and (**b**) distribution of BC incidence location $\mathop{{\boldsymbol{s}}}\limits^{{\boldsymbol{\frown }}{}}$ after coordinate transformation $\mathop{{\boldsymbol{s}}}\limits^{{\boldsymbol{\frown }}{}}={\boldsymbol{s}}-{\boldsymbol{\upsilon }}t$. Maps were generated using ArcGIS 9.3. URL link: http://desktop.arcgis.com/zh-cn/desktop/.

**Figure 6:**
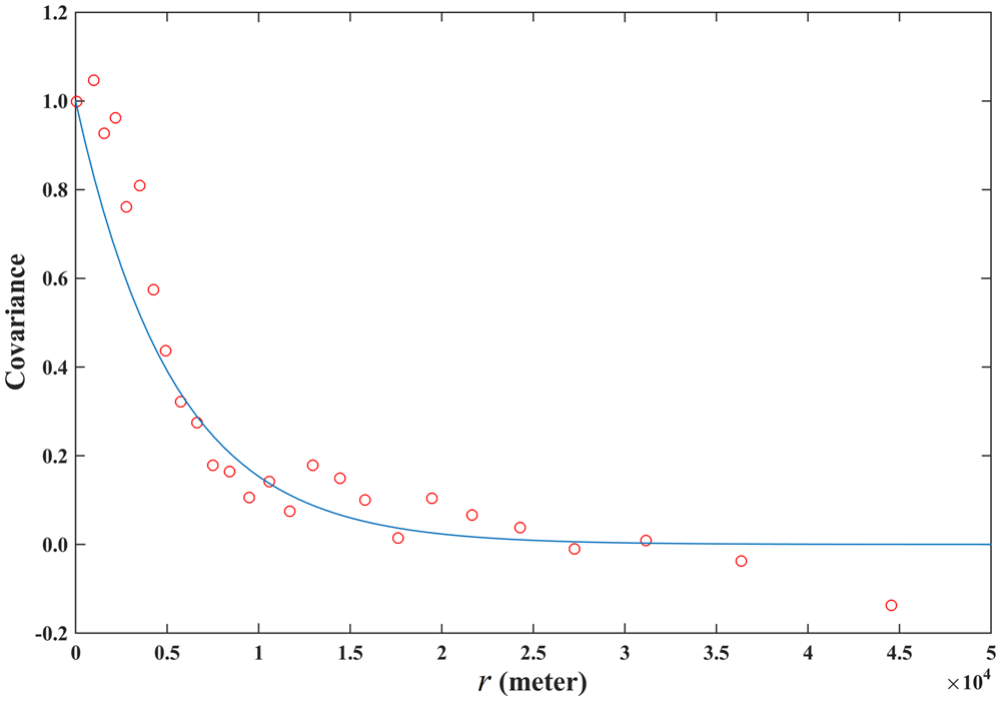
Empirical covariance (red dots) and theoretical covariance model (continuous line) of the traveling BC incidence distribution in *R* ^2^.

The empirical covariance ${\hat{c}}_{BC}({\boldsymbol{h}},\tau )$ of the original space-time *BC*(***s***, *t*) incidence is plotted in Fig. 4. The spatiotemporal covariance function *c*_*BC*_(***h***, *τ*) of Eq. (3) was selected as the theoretical *BC*(***s***, *t*) covariance model (also shown in Fig. 4) and subsequently fitted to the empirical covariance. It was found that the *c*_*BC*_(***h***, *τ*) values were high close to the space-time origin, but declined very quickly with increasing |***h***| and *τ* values. The covariance value is almost zero for |***h***| = *ε*_*s*_ > 10 *km* and *τ* = *ε*_*t*_ > 2 *yrs*. The short spatial correlation range contributed to a higher spatial variability in incidence distribution. After the coordinate transformation $\mathop{{\boldsymbol{s}}}\limits^{{\boldsymbol{\frown }}{}}={\boldsymbol{s}}-{\boldsymbol{\upsilon }}t$, the distribution of BC incidence locations is shown in Fig. 5, and the empirical covariance of $BC(\mathop{{\boldsymbol{s}}}\limits^{{\boldsymbol{\frown }}{}})$ is plotted in Fig. 6 (red dots). The comparison of Figs 1c and 5 revealed an interesting feature of the STP dimensionality reduction notion. In particular, Figs 1c and 5 show plots of, respectively, the original BC incidence coordinates *s*_1_, *s*_2_ (in *R* ^2^ × *T*), and of the transformed BC incidence coordinates ${\mathop{s}\limits^{\frown {}}}_{1}={s}_{1}-\upsilon t$, ${\mathop{s}\limits^{\frown {}}}_{2}={s}_{2}-\upsilon t$ (in *R* ^2^). Clearly, in the original domain the coordinates *s*_1_ and *s*_2_ are entirely uncorrelated, whereas in the transformed domain the coordinates ${\mathop{s}\limits^{\frown {}}}_{1}$ and ${\mathop{s}\limits^{\frown {}}}_{2}$ are strongly correlated. The exponential function of Eq. (8) was selected as the theoretical ${c}_{BC}(\mathop{r}\limits^{\frown {}})$ covariance model (continuous line in Fig. 6). Compared to the BC incidence covariance *c*_*BC*_(***h***, *τ*), the transformed incidence covariance ${c}_{BC}(\mathop{r}\limits^{\frown {}})$ revealed a much stronger spatial correlation among the transformed $BC(\mathop{{\boldsymbol{s}}}\limits^{{\boldsymbol{\frown }}{}})$ data. The correlation range was about 20 *km*, two times larger than that of *c*_*BC*_(***h***, *τ*). Also, the ${c}_{BC}(\mathop{r}\limits^{\frown {}})$’s slope at the space origin was considerably lower compared to that of *c*_*BC*_(***h***, *τ*), meaning that the spatial variation of $BC(\mathop{{\boldsymbol{s}}}\limits^{{\boldsymbol{\frown }}{}})$ was much more continuous and smoother than of *BC*(***s***, *t*).

STP simplifies the study (modeling and estimation) of the BC incidence distribution by transferring it into a domain of lower dimensionality (i.e., from *R* ^2^ × *T* to *R* ^2^). Accordingly, the STP technique demonstrated a superior performance compared to the mainstream STOK technique of space-time incidence mapping. A comparison of the estimation accuracy of the two techniques (STP vs. STOK) is shown in Table 2. Three commonly used accuracy indicators, mean error (ME), mean absolute error (MAE), and root mean square error (RMSE), were used to test the numerical accuracy of the estimation results. Obviously, the STP technique generated considerable more accurate BC incidence estimates than the STOK technique: the ME, MAE and RMSE ffigvalues of the BC estimates generated by the STP technique were much lower (−2.99, 17.39 and 21.62/100,000 respectively) than those generated by the STOK technique (5.61, 28.26 and 44.41/100,000, respectively).

**Table 2:** Comparison of the BC incidence estimations accuracy of the STP and STOK techniques.

	ME^a^	MAE^b^	RMSE^**c**^
*STOK*	5.61	28.26	44.41
*STP*	−2.99	17.39	21.62

^a^Mean error (1/100,000).

^b^Mean absolute error (1/100,000).

^c^Root mean square error (1/100,000).

**Figure 7:**
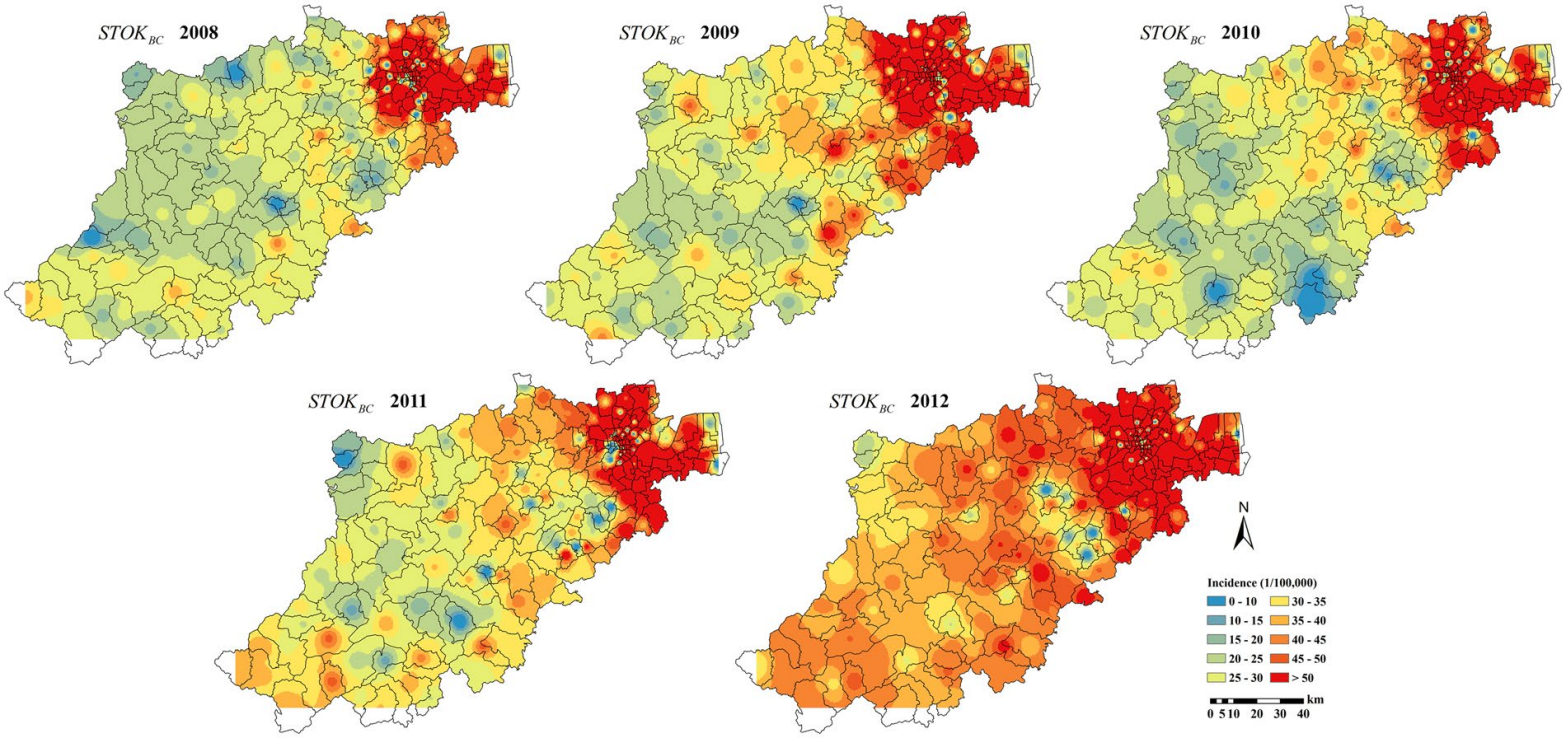
STOK estimation maps of the BC incidence distribution in Hangzhou city during 2008–2012. Maps were generated using ArcGIS 9.3. URL link: http://desktop.arcgis.com/zh-cn/desktop/.

**Figure 8:**
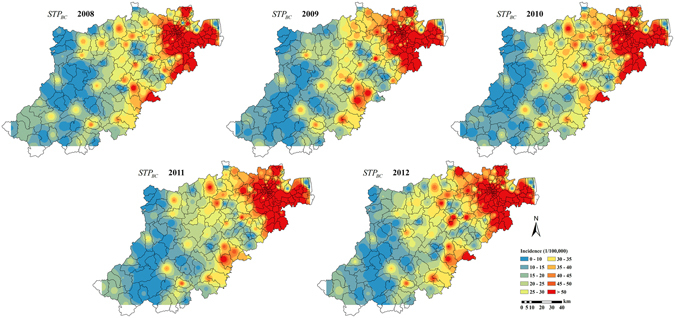
STP estimation maps of the BC incidence distribution in Hangzhou city during 2008–2012. Maps were generated using ArcGIS 9.3. URL link: http://desktop.arcgis.com/zh-cn/desktop/.

**Figure 9:**
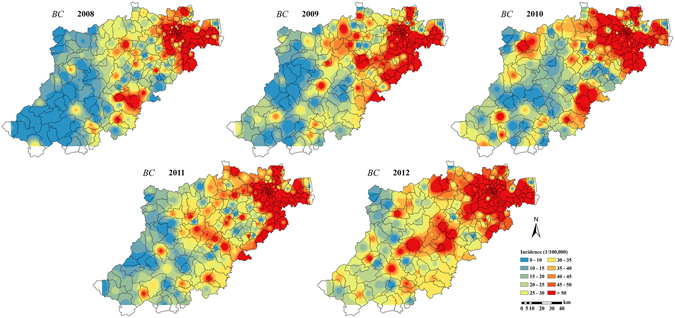
The original distribution of BC incidence. Maps were generated using ArcGIS 9.3. URL link: http://desktop.arcgis.com/zh-cn/desktop/.

This difference in accuracy in favor of the STP technique was clearly observed in the corresponding BC incidence estimation maps of Hangzhou city during the period 2008 to 2012. The BC incidence estimation maps produced by the STOK technique, map_*STOK*_(***s***, *t*), and by the STP technique, map_*STP*_(***s***, *t*), are shown in Figs 7 and 8, respectively. Compared to the actual distribution of BC incidence (Fig. 9), the estimated map_*STOK*_(***s***, *t*) tends to over-estimate the BC incidence in the southwest low incidence regions and to underestimate it in the northeast high incidence regions. The map_*STP*_(***s***, *t*), on the other hand, was found to be closer to the actual BC distribution during the study period, and also much more stable. Specifically, the map_*STP*_(***s***, *t*) exhibited a definite trend from the southwest low incidence region to the northeast high incidence region, which is also the trend of the actual incidence distribution. Hence, the STP technique provided a more informative and realistic representation of the actual BC distribution in Hangzhou city during the period 2008–2012.

**Figure 10:**
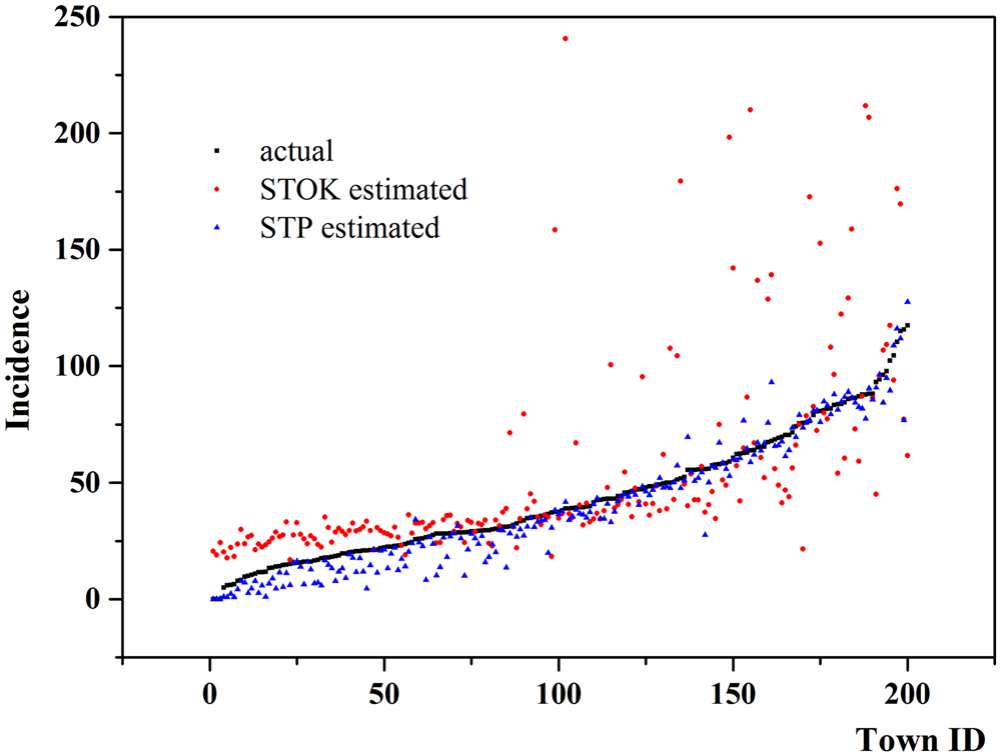
Plots of the 5-year averaged BC incidence at 1000 town points for the period 2008–2012: (**a**) actual BC incidence values (black points), (**b**) STOK estimated incidence values (red points), and (**c**) STP estimated incidence values (blue points). Incidence per 100,000 people.

An additional accuracy test is shown in Fig. 10, which also suggested that the STP technique performed considerably better than the mainstream STOK technique in estimating the BC incidence distribution. Specifically, the 5-year averaged BC incidence of the actual data together with the STP and STOK estimates in 200 townships are plotted in Fig. 10. The town ID is denoted from 1 to 200 with ascending order of actual BC incidence data. Just like the BC incidence distribution presented in the map_*STOK*_(***s***, *t*), the STOK estimation tends to over-estimate the BC incidence in low incidence regions and underestimate them in high incidence regions. Moreover, the STOK generated unrealistic BC incidence estimates in the middle and high incidence regions. On the contrary, the STP estimates provided an almost perfect fit to the actual BC incidence values in the middle and high incidence regions, and they slightly underestimated the BC incidence values in the low incidence regions.

Lastly, regarding the computational cost of the two techniques, a typical computer time of the STOK technique was 627 secs and of the STP technique 463 secs (i.e., an about 26.2% cost reduction).

## Discussion

Space-time estimation and mapping techniques can improve our understanding of disease distribution and offer valuable information for risk assessment and health management purposes. The present study focused on the application of the STP technique in the modeling and estimation of space-time BC incidence in Hangzhou City. Methodologically, the STP is a novel technique that is based on the three-fold idea of “Transform-Solve-Backtransform”. In particular, the STP (*a*) first reduces the study of a complex three-dimensional data set to that of a two-dimensional data set (a reduction with considerable modeling and computational advantages, as discussed earlier), (*b*) then solves the BC incidence estimation problem using only the transformed incidence data set in the reduced dimensionality spatial (*R* ^2^) domain, and (*c*) finally it backtransforms the results to the original space-time domain (*R* ^2^ × *T*). Otherwise said, the STP idea is to temporarily “compress” the time information at the transformation stage, solve the BC mapping problem in the much simpler domain of the “compressed” time data, and then release the “compressed” time data information at the backtransformation (final) stage. In this setting, the “compressed” domain (*R* ^2^) is a transitional stage whose purpose is to simplify the BC data analysis.

STP was also compared to the well-known STOK technique of space-time disease mapping. STOK is a mainstream technique that has been extensively used to estimate the distribution of attributes across space-time, such as disease mortality, human exposure and environmental health indicators^5, 28, 29^. A few studies have used this technique to estimate BC incidence distributions, but they were based on rather small data sets. Given the availability of a sufficiently large BC incidence data set in the Hangzhou city area (a total of 8784 cases at 200 towns during 5 years), we employed the STOK technique to estimate the spatiotemporal distribution of BC incidence in Hangzhou city.

It has been reported in the relevant literature that the implementation of the STOK technique in practice experiences certain difficulties, including the rather complicated process of selecting an adequate spatiotemporal covariance model and the associated parameter estimation, especially when non-separable covariance models are involved, and also, the adequate determination of spatiotemporal distances (metrics), which is usually not an easy matter^18^.

Compared to the STOK technique, the STP technique is more accurate, easier to implement, and also more workable with the software libraries available. After the dimensionality of the BC incidence distribution has been reduced from three (space-time) to two (space only), BC incidence estimation becomes considerably easier and efficient, including locational coordinate arrangements and covariance determination. Based on the BC incidence correlation plots obtained in the *R* ^2^ × *T* and *R* ^2^ domains, see Figs 4 and 6, respectively, it was found that covariance modeling and parameter estimation is generally much easier in the latter than in the former domain. Figure 4 presents a two-dimensional plot of the BC incidence covariance as a function of two distinct arguments, space and time, which have different effects on BC incidence variation (physically, distance in space differs drastically from “distance” in time, and the determination of composite space-time distances is usually a complicated process)^25^. There is an imbalance in the information content associated with the spatial vs. the temporal dimension (a common case is a geographically large study area with a short study period). The above facts often make it much harder to select a spatiotemporal covariance model that represents adequately the composite space-time variation structure of BC incidence. Complexity varies with the form of the selected theoretical space-time covariance model to be fitted to the data. For example, it is easier to specify the parameters of a multiplicative (product) space-time model on the basis of the available data, and much more difficult to do the same for an additive (summation) space-time covariance model. On the other hand, Fig. 6 is a unidimensional plot of the transformed BC covariance used by STP, the specification of which does not involve any of the complications mentioned above. Naturally, it is always easier to select an adequate covariance model and specify its parameters in the *R* ^2^ domain than in the *R* ^2^ × *T* domain.

Based on the BC incidence maps obtained in Hangzhou city from 2008 to 2012 (Fig. 8), it was found that the incidence distribution in the area is temporally stable and spatially heterogeneous, and the incidence map_*STP*_(***s***, *t*) revealed an increasing incidence trend from the southwest to the northeast region. This result is consistent with previous studies^21^, which investigated the heterogeneity of BC incidence in the time, the space and the composite space-time domains with the help of Analysis of Variance (ANOVA), Poisson Regression and Space-time Scan Statistics. Many factors may lead to this heterogeneity, and the key cause may be urban sprawl^30, 31^. The recent economic development in towns and subdistricts areas makes it easier for residents to obtain health care, so that more early-stage BC cases are diagnosed in these areas. In addition, economic development implies higher pollution (such as heavy metals and dioxins), which may lead to higher BC risk^32, 33^. The northeast high BC incidence area provided the empirical means to calculate the BC incidence velocity vector in Eqs (1) and (2). Generally, a more accurate calculation of the velocity vector based on the available information leads to a more accurate BC incidence estimates generated by the STP technique.

In sum, by transforming the space-time domain (*R* ^2^ × *T*) into a spatial domain (*R* ^2^) of reduced dimensionality, the STP technique eliminates a number of theoretical and practical difficulties and complexities, such as follows:

i. The STP avoids some serious space-time disease modeling problems, like the determination of the space-time metric (“distance”) in a way that blends space and time but also accounts for the fact that space and time have very different physical properties.
ii. The STP reduces considerably the computational effort and the associated approximations it introduces (i.e., fewer computations imply fewer numerical approximations).
iii. The transformation introduced by STP enhances the composite space-time correlation structure of disease incidence (e.g., the correlation lags between “data-to-data” and “data-to-estimation” points become shorter in the reduced dimensionality domain, and can be computed much easier and accurately than the space-time lags in the original higher dimensionality domain).
iv. A large part of the uncertainty associated with mainstream space-time techniques, like the STOK technique, is due to the errors involved (*a*) in the determination of the space-time incidence cross-correlations, (*b*) the specification of the physical differences between spatial and temporal variations, and (*c*) the selection of adequate theoretical incidence covariance models (including model parameter specification). All these errors are avoided in the case of the coordinate transformation introduced by the STP technique.

It was also found that the BC incidence estimates in the transition zone between the high-incidence city center and the low-incidence rural areas are a little lower than the actual BC values. The same situation was also observed in the scatter diagram of Fig. 10, where the BC values are slightly under-estimated by STP. One explanation may be that, as mentioned earlier, the velocity vector expresses average space-time BC spread. Another factor may be the existence of a considerable number of zero-incidence regions (showed in Fig. 2), which may affect data normalization and the corresponding BC incidence covariance^34^. Nevertheless, comparing to the STOK estimation map, which seriously overestimates low-incidence and under-estimates BC incidence in the mid- and high-incidence areas, the STP maps provide much better representations of the actual BC situation, especially in the middle- and high-incidence regions, where it matters most.

We notice that the STP technique has been also successfully used in our earlier work^18^ to study simulated mortality rates of an infectious disease. In the present work, we have used real BC incidence data to show that the STP technique also performs very well in the case of real-world noninfectious diseases (with pathogenic mechanism and spatiotemporal distribution that are totally different than those of an infectious disease). Moreover, the successful application of STP in the present infectious disease study implies that the STP could be used to study the space-time distribution of other important diseases (lung cancer, stomach cancer etc.).

In addition, the STP technique could be combined with other advanced spatiotemporal theories and models, such as the Bayesian maximum entropy (BME) theory. BME has different but complementary objectives than the STP technique, namely, it integrates various kinds of knowledge bases, such as scientific laws, empirical relationships, auxiliary information, hard and soft data of varying uncertainty levels. Naturally, a combination of the BME properties with the STP features could lead to a method that incorporates the advantages of both component methods. For example, in the present study only exact BC incidence data (i.e., data with a negligible uncertainty level, also called *hard data* in the BME terminology) was used. Considering that BC incidence is strongly correlated with socioeconomic status^35^, lifestyle, and environment exposure^36, 37^, etc. (all of them belong to the *soft data* category in the BME terminology), the combination of STP and BME could be produce more accurate and informative BC incidence predictions, and should be the topic of a future research.

By way of a summary, BC is one of the highest-incidence cancers among females. The use of the STP technique relies on the inter-connection between BC incidence, space, time, and incidence spread velocity, which can be specified in a self-consistent manner so that the STP technique can make it much easier and more realistic to estimate space-time BC incidence distributions than other mainstream techniques, and to detect potential relationships with other human exposure and environmental risk factors, thus providing valuable information for BC control and prevention.
